# Impact of Empathic Concern on Prosocial Behavior in Gain and Loss Contexts: Evidence from Event-Related Potential

**DOI:** 10.3390/brainsci14040400

**Published:** 2024-04-19

**Authors:** Xi Luo, Taowen Yu, Min Tan, Yiping Zhong

**Affiliations:** 1 Department of Psychology, School of Education Science, Hunan Normal University, Changsha 410081, China; luoxi89@hnfnu.edu.cn (X.L.); mintan2021@163.com (M.T.); 2 School of Physics and Chemistry, Hunan First Normal University, Changsha 410205, China; 3 Cognition and Human Behavior Key Laboratory of Hunan Province, Changsha 410081, China; 4 Hunan Key Laboratory for Children’s Psychological Development and Brain and Cognitive Sciences, Changsha 410205, China; 5 Department of Psychology, Changsha Normal University, Changsha 410100, China; 202238061017@mail.bnu.edu.cn

**Keywords:** empathic concern, gain/loss context, prosocial behaviors, event-related potential (ERP)

## Abstract

This study employs event-related potential (ERP) to examine the impact of empathic concern on prosocial decision-making with costs in both gain and loss contexts. In this experiment, participants can choose between two types of lottery tickets and pay lottery money to help the target person gain more money or lose less money. The behavioral results showed that regardless of the context of the decision (financial loss or gain), participants tended to help individuals who had induced high empathic concern. ERP results show that compared to the low-empathic-concern condition, the high-empathic-concern condition induced greater P3 amplitude in the gain context. However, this change in P3 amplitude caused by empathic concern did not occur in the context of loss. These findings indicate that empathic concern has different psychological mechanisms that moderate prosocial behavior in gain and loss contexts.

## 1. Introduction

Prosocial behavior refers to benefiting other individuals, groups, or society as a whole [1]. In daily life, people often sacrifice certain personal interests, such as time and money, when engaging in prosocial behavior [2,3]. Previous studies show that empathy is a significant driving force for prosocial behavior [4]. Batson’s “empathy altruism” hypothesis suggests that when others are in trouble, individuals will produce a series of emotions, including empathic concern, sympathy, and pity, and generate altruistic motivation to relieve their plight, thus exhibiting prosocial behaviors [5]. Empathic concern, or state empathy, is an emotional and motivational state characterized by helping and promoting the welfare of others [6,7,8]. Previous studies have shown that inspiring individual empathy can lead to more prosocial behaviors in others, such as cooperative behavior and donation assistance [9,10]. Studies using neuroimaging methods show that the activation of the ventral striatum is enhanced when prosocial actions toward empathic goals are undertaken [11,12]; that is, humans are most concerned with the welfare of those they empathize with. This is probably because the emotional rewards are greater when individuals engage in prosocial behavior toward those they are close to [13,14].

In real life, the outcome of helping others may help individuals gain money or avoid losses. However, most studies that have explored the impact of trait empathy on prosocial behavior infer that helping others helps earn more money. Notably, people have different psychological feelings and behavioral responses in the face of loss and gain. Specifically, the loss context can cause greater subjective effects than the gain context and induce loss aversion, which results in a large behavioral difference. However, to the best of our knowledge, research on the impact of empathic concern on prosocial behavior in the context of loss is insufficient. Previous studies have shown that individuals tend to associate losses with unfairness. Therefore, in the field of losses, individuals place greater emphasis on fairness [15,16,17]. Neuroimaging research also shows that individuals act in accordance with the principle of fairness in the loss context and activate fairness-related brain regions, such as the insula, dorsolateral prefrontal cortex, and medial orbitofrontal cortex [16,18,19,20]. Social distance and state empathy were positively correlated, and individuals were more likely to empathize with people at close social distance [21,22]. Some studies employed event-related potential (ERP) technology and further found that people have similar helping behavior and P300 amplitude for friends and strangers in the context of loss [23], and that people are more inclined to follow the principle of fairness. Yamamoto and Takimoto highlighted that fairness can inhibit empathy-induced prosocial behavior [24]. This suggests that individuals are likely to help others regardless of their empathic concern for others in situations of loss. So far, there have been few studies that directly compare the impact of empathic concern on prosocial behavior in different (e.g., loss and gain) contexts.

Previous studies have several limitations in the behavioral and neuroimaging aspects of the discrete cognitive processes that trigger prosocial decision-making [25]. ERP can overcome the limitations of behavioral and neuroimaging studies in understanding the time course underlying prosocial decision-making [26]. Therefore, we used ERP technology to examine the impact of empathic concern on prosocial behavior in gain and loss contexts. P200 is a positive component that appears in the prefrontal brain region about 200 ms after the presentation of processing stimuli and is moderated by emotional responses [27,28,29]. In previous studies, the P200 effect was mainly reflected in the processing of empathy for pain; empathy for pain is a special form of empathy [30]. P200 is an emotional response to the pain of others, and the stronger the emotional response, the greater the amplitude induced [31,32]. P300 is typically present in the central and parietal regions of the brain as a positive wave that peaks within 300–600 ms after stimulus onset, and is an important psychological component associated with prosocial behavior [33]. It also reflects the allocation of attentional and motivational or affective salience [20,34]. In the case of assistance, the amplitude of P300 increases with the individual’s perceived needs, indicating that it is sensitive to the significance of motivation and emotion [33,35]. Previous studies show that empathy can effect changes in P300 during the decision-making process [36,37], reflecting changes in the level of emotional motivation. Empathy increases prosocial–emotional motivation when making decisions regarding empathic targets, resulting in a larger P300 amplitude [7,38,39].

In conclusion, individuals have different neural responses to strangers with different levels of empathic concern in gain and loss situations, which is reflected in P200 and P300. Particularly, empathic concern increases an individual’s attentional and motivational or affective levels of prosocial behavior, and enhances P200 and P300 amplitude [40,41]. Therefore, we assume that, in the gain context, individuals make more prosocial decisions and induce a larger P200 and P300 when faced with underprivileged people. However, individuals follow the principle of fairness in the context of losses [14,19,33], and the amplitudes of P200 and P300 do not vary with empathic concern.

## 2. Materials and Methods

### 2.1. Participants and Experimental Design

We used G*Power 3.1 software to calculate the sample size required to ensure sufficient statistical efficacy, and the results showed that 24 participants were able to meet the minimum sample requirement for this experiment [42]. We recruited 36 college students (13 male, mean age = 18.89, *SD* = 1.09), all of whom were right-handed, had no history of mental disease or brain injury, and had normal or corrected vision. Before the start of the experiment, all participants signed informed consent forms, and after the experiment, the participants received corresponding experimental compensation. The experiment used a 2 (empathic concern: low empathic concern vs. high empathic concern) × 2 (contexts: gain context vs. loss context) within-participant factorial design.

### 2.2. Empathic Concern Manipulation

The receiver who was in the high-empathic-concern condition was a student from a remote poverty region. The receiver who was in the low-empathic-concern condition was a general student studying in a normal urban school. Participants were asked to fully read the text describing the family status and living conditions of the underprivileged and general students to induce a state of empathic concern. Both receivers were set up as strangers to the subjects [43]. After reading the description statements of two receivers, the participants were asked to what degree they empathized with the underprivileged and general students and what their empathic concern towards underprivileged and general students was (a seven-point Likert scale rating) [24]. We used the average score of these two questions as the empathic concern manipulation check. The higher the score, the higher the degree of empathic concern.

### 2.3. Choosing Lottery Task

The lottery task we used was adapted from Everett et al.’s research on prosocial behavior [44], which is consistent with previous studies [26]. This study used a lottery selection task to explore prosocial behavior. In the experiment, each participant is required to complete a lottery task, in which they play the role of the “decider” and make decisions for the target person who was assigned the role of a “receiver” (i.e., an underprivileged student or a general student). At the beginning of the experiment, participants received starting money and were asked to choose between two lottery tickets that were relatively expensive compared to the starting money, with different colors representing gains and losses. For example, when the lottery displayed on the screen was green, if the participants chose the lottery on the left, their starting money would decrease by CNY 0.1, and the probability of the target person receiving CNY 0.5 was 80%, but if the participant chose the lottery on the right side, the probability of the target character receiving CNY 0.5 was only 20% without a decrease in starting money. When the red ticket was presented, participants chose the ticket on the left, the starting money decreased by CNY 0.1, and the probability of the target person losing CNY 0.5 was 20%. Alternatively, if the participant chose the ticket on the right, the starting money did not decrease but the probability of the target person losing CNY 0.5 was 80%. This study balanced the colors representing loss or gain contexts in the experiment. In the context of gain, participants paid more money to increase the probability of the target to earn more money. In the context of loss, participants paid more money to reduce the probability of the receiver’s loss.

Participants were paid CNY 30 to participate in the experiment, but how much money they ultimately received depended on their choice of task. Specifically, participants in each round of the experiment had to decide whether to spend CNY 0.05 to help others, and if they chose to spend CNY 0.05 to help others, then CNY 0.05 was deducted from the CNY 30 participation fee, and all choices accumulated until the end of the experiment. The final participation fee was determined by deducting the participation fee according to the proportion of the participant’s help in the task (*M* = 25.33, *SD* = 2.54).

### 2.4. Procedure

**Figure 1:**
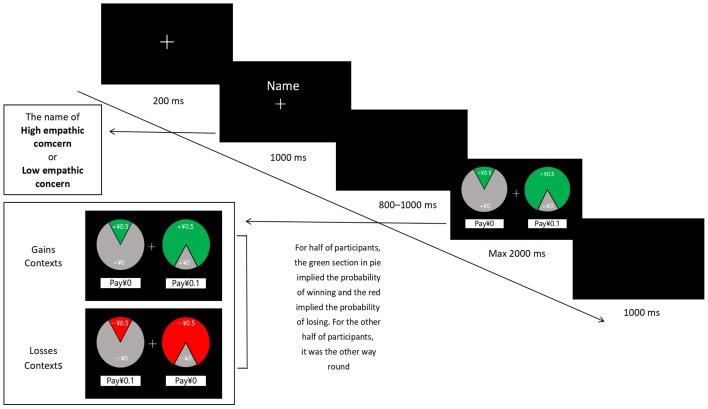
The timeline of a trial. At the beginning of each trial (round) of the experiment, a fixation cross is presented in the center of a black background for 200 ms. To demonstrate the “Receiver” in this experiment, a 1000 ms “Receiver” name (i.e., high or low empathic concern) is presented above the fixation cross. After an interval of 800–100 ms, two lottery tickets are presented on the left and right sides of the fixation cross. In the context of the gain experiment, if participants choose the lottery on the left (right) side, they will not have to pay, and there is a probability of 20% that the receiver will receive CNY 0.5. If participants choose the lottery ticket on the right (left) side, their starting money will be reduced by CNY 0.1 and the receiver has an 80% probability of receiving CNY 0.5. In the context of the loss experiment, if the subjects choose the lottery ticket on the left (right) side, their starting amount will be reduced by 0.1, while the receiver has a 20% probability of losing 0.5. If the subject chooses the lottery on the right (left) side, they will not have to pay, and the receiver has an 80% probability of losing CNY 0.5. Under two experimental conditions, this study balanced the appearance of lottery tickets and the colors representing profit and loss. Additionally, to eliminate the influence of color confusion in the ERP results, the red part of the lottery, presented to half of the participants, represents the probability of gains, while the green part represents the probability of losses. For the other half of the participants, green represents profit and red represents loss. The presentation time of the decision-making interface is 2000 ms, and the participants are required to press the “F” or “J” key to make a selection for the lottery. After completing the selection within 1000 ms, they enter the next trial. If the subject does not make a decision within 2000 ms, they will proceed directly to the next trial.

E-Prime software (Version 3.0) was used to present the experimental stimuli and record behavioral data. As shown in Figure 1, a fixation cross was displayed in the center of the screen for 800–1200 ms before each trial, followed by a black background of 200 ms. The name of the receiver (the poor student or general student) appeared, followed by a black background of 800–1000 ms, after the lottery task was chosen, and the two lottery tickets were displayed on both sides of the central screen. Two lottery tickets were presented on the screen for 2000 ms, and participants were asked to press the “F” or “J” keys, respectively, to select the corresponding lottery tickets. If the participants did not respond within 2000 ms, the next trial was conducted. If the participants made a timely choice, the next trial started after a time interval of 1000 ms. There were four conditions in the experiment, with 60 trials per condition. To ensure an understanding of the task rules, each participant was instructed to perform the lottery task 12 times before the formal experiment.

### 2.5. EEG Recording and Analysis

The 64-channel EEG recording system by Brain Products was used, and the electrodes were fixed on an electrode cap in an internationally recognized 10–20 format. In this study, Fz was selected as the reference electrode, and offline analysis was switched to bilateral mastoid reference. The grounding electrode was connected to the center of the forehead. The electrode located in the middle of the upper and lower eye sockets of the left eye recorded vertical eye electrical activity (VEOG), while the electrode located 1 cm outside the left and right eye corners recorded horizontal eye electrical activity (HEOG). The impedance between each electrode and the scalp was maintained below 5 kΩ. When recording continuously, the filtering bandpass was 0.5~100 Hz, the sampling rate was 500 Hz, and EEG data were preprocessed using the EEGLAB toolbox of MATLAB R2016a software. The original EEG data were segmented from 200 ms before the stimulus (lottery choice) to 800 ms after it. We filtered the EEG data to remove data segments containing high-amplitude noise, such as large-amplitude movements. Other consistent artifacts were further eliminated using an Independent Component Analysis (ICA) algorithm. Apparent artifacts with amplitudes exceeding ±100 μV were eliminated. The superimposed average was then calculated for different conditions. Based on the topographic distribution of the ERP components and previous research, N200 selected 220–300 ms as the time window for analysis, 160–220 ms as the time window for P200, and 300–400 ms as the time window for P300. The N200 was calculated using 6 electrode sites (F1, F2, FZ, FC1, FC2, FCZ), the P200 was calculated using 7 electrode sites (FC4, FCZ, F4, F1, FZ, CZ, C4), and the P300 was calculated using 9 electrode sites (C1, C2, CZ, P1, P2, PZ, CP1, CP2, CPZ).

## 3. Results

### 3.1. Empathic Concern Manipulation Checks

Participants self-reported that the empathic concern scores between high- and low-empathic-concern targets differed significantly, with *t*(35) = 11.689, *p* < 0.001, Cohen’s d = 1.948, indicating that participants had stronger empathic concern toward high-empathic-concern targets (11.67 ± 1.773) than low-empathic-concern targets (5.67 ± 2.798). According to existing research, the manipulation of empathic concern is effective.

### 3.2. Behavioral Results

**Figure 2:**
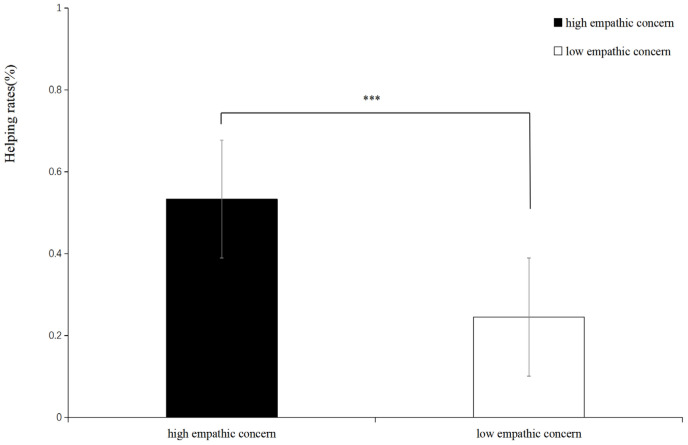
The bar graph of helping rates in each condition. *** *p* < 0.001.

Because the gender of the participants was not balanced, in order to control the effect of gender, we included gender as an independent variable in all analyses, and repeated-measures ANOVA was conducted using the helping rate as the dependent variable. The results showed that the main effect of empathic concern was significant (*F*(1,35) = 45.025, *p* < 0.001, *η_p_*^2^ = 0.563), and the help ratio of the high-empathic-concern condition (*M* = 0.533) was significantly higher than that of the low-empathic-concern condition (*M* = 0.245). Other effects were insignificant (*p*s > 0.05) (see Figure 2).

Repeated-measures ANOVA was conducted using decision time as the dependent variable. All results were insignificant (*p*s > 0.05).

### 3.3. ERP Results

#### 3.3.1. N200

**Figure 3:**
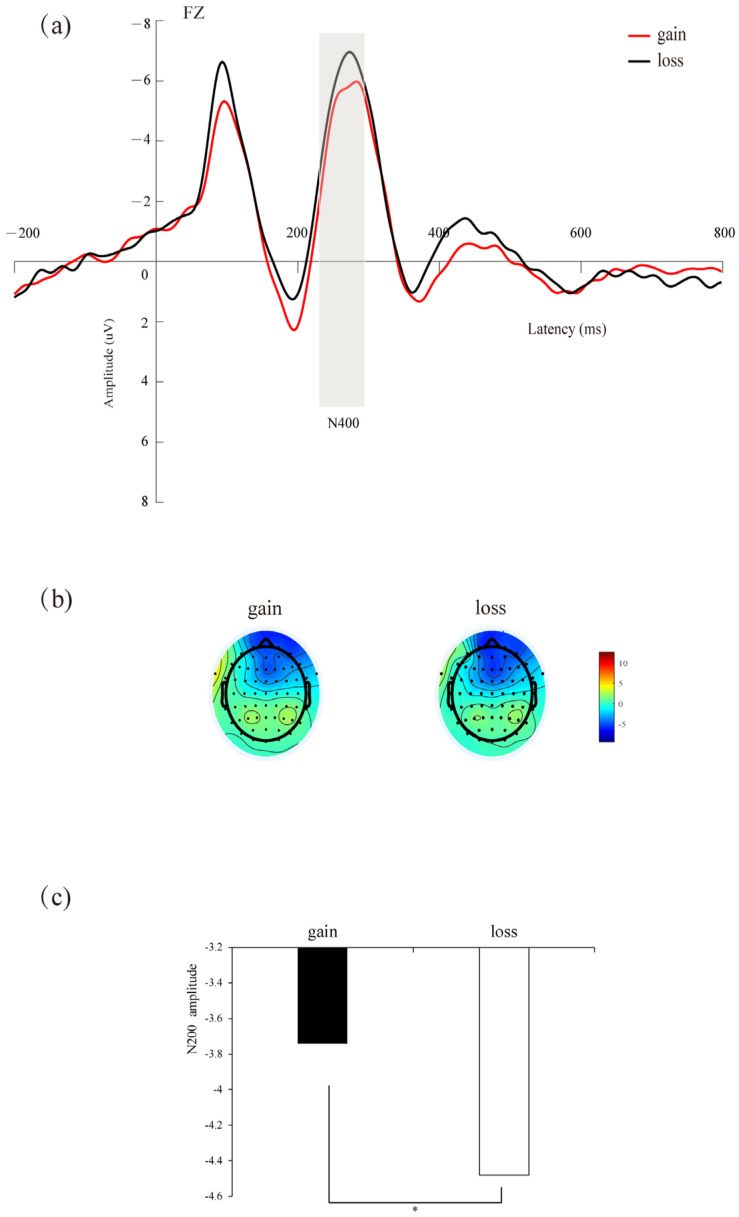
(**a**) Amplitude at Fz electrode site. The gray bar indicates the time window of the N200 (230–300 ms) used for statistical analysis. (**b**) Scalp maps of different waveforms in the 230–300 ms time window. (**c**) Bar graphs of the mean N200 values associated with each condition. * *p* < 0.05.

Repeated-measures ANOVA showed that the main effect of context was significant (*F*(1,34) = 18.236, *p* = 0.029, *η_p_*^2^ = 0.132), and the N2 amplitudes induced by the loss context (*M* = −4.477 μV) were larger than the gain context (*M* = −3.736 μV) (see Figure 3). No other significant impacts were identified (*p*s > 0.05).

#### 3.3.2. P200

**Figure 4:**
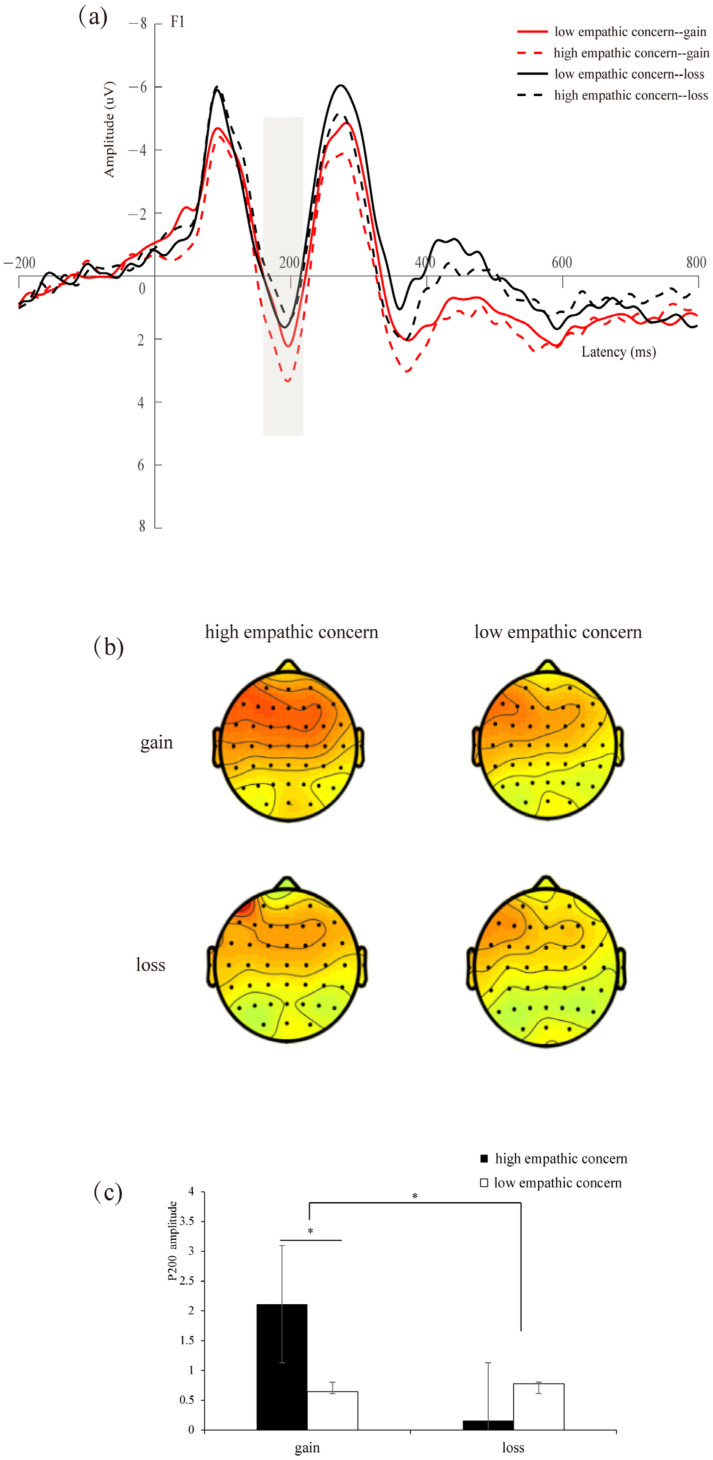
(**a**) Amplitude at F1 electrode site. The gray bar indicates the time window of the P200 (160–220 ms) used for statistical analysis. (**b**) Scalp maps of different waveforms in the 160–220 ms time window. (**c**) Bar graphs of the mean P200 values associated with each condition. * *p* < 0.05.

Repeated-measures ANOVA showed that the main effect of context was significant (*F*(1,34) = 12.426, *p* = 0.001, *η_p_*^2^ = 0.268), and the P200 amplitudes induced by the gain context (*M* = 1.373 μV) were larger than those in the loss context (*M* = 0.459 μV). The interaction between empathic concern and context was significant (*F*(1,34) = 10.292, *p* = 0.003, *η_p_*^2^ = 0.232), and the simple effect found that the difference between the high- and low-empathic-concern conditions was significant in the gain context (*F*(1,34) = 6.142, *p* = 0.018, *η_p_*^2^ = 0.153). However, there was no significant difference between the high- and low-empathic concern conditions in the loss context (*F*(1,34) = 0.782, *p* = 0.383, *η_p_^2^*= 0.022) (see Figure 4). No other significant impacts were identified (*p*s > 0.05).

#### 3.3.3. P300

**Figure 5:**
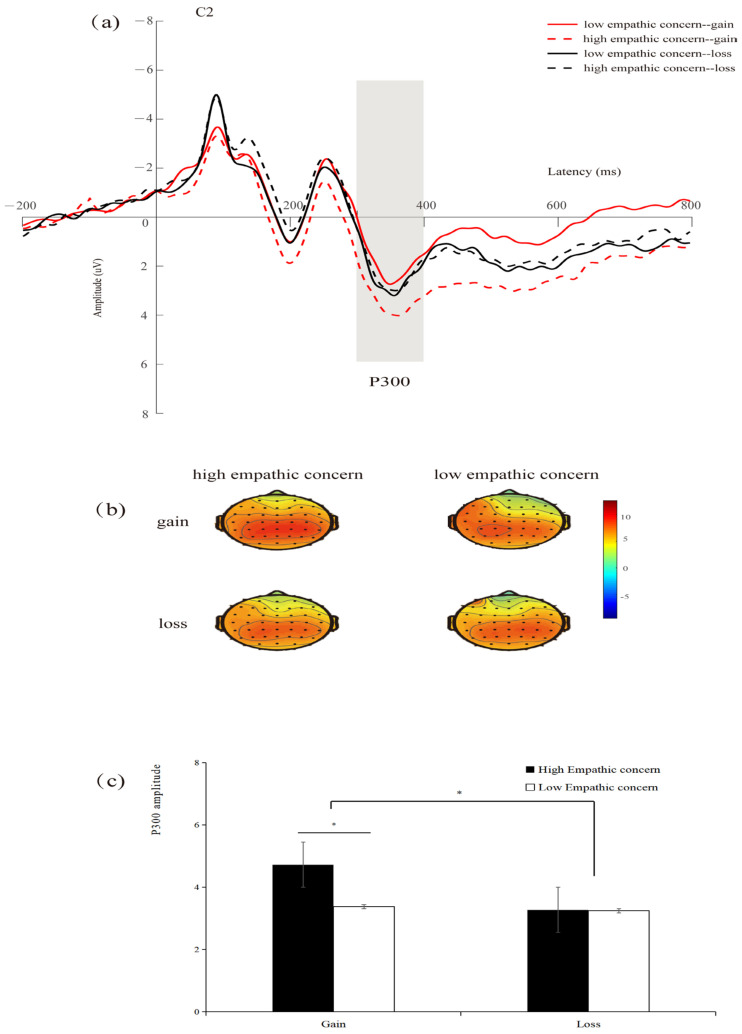
(**a**) Amplitude at C2 electrode site. The gray bar indicates the time window of the P300 (300–400 ms) used for statistical analysis. (**b**) Scalp maps of different waveforms in the 300–400 ms time window. (**c**) Bar graphs of the mean P300 values associated with each condition. * *p* < 0.05.

Repeated-measures ANOVA showed that the main effect of context was significant (*F*(1,34) = 6.853, *p* = 0.013, *η_p_*^2^ = 0.168), and the P300 amplitudes induced by the gain context (*M* = 4.052 μV) were larger than those in the loss context (*M* = 3.259 μV). The interaction between empathic concern and context was significant (*F*(1,34) = 4.804, *p* = 0.035, *η_p_*^2^ = 0.124), and the simple effect found that the difference between the high- and low-empathic-concern conditions was significant in the gain context (*F*(1,34) = 7.169, *p* = 0.011, *η_p_*^2^ = 0.174). However, there was no significant difference between the high- and low-empathic-concern conditions in the loss context (*F*(1,34) = 0.002, *p* = 0.966) (see Figure 5). No other significant impacts were identified (*p*s > 0.05).

## 4. Discussion

This study examines the neural mechanisms of empathic concern in prosocial decision-making in different contexts, using a lottery selection task where participants provide assistance to others in loss and gain contexts. The behavioral results confirm the hypothesis that individuals are more willing to sacrifice their money to provide assistance to a high-empathic-concern target. Consistent with our prediction, in terms of the neural response, the average amplitude of P200 and P300 increases when providing assistance to high-empathic-concern objects; however, it is worth noting that this change only occurs in the gain context. These findings suggest that empathic concern can modulate the late stages of prosocial decision-making. When playing games against high-empathic-concern objects, individuals exhibited an enhanced P200 and P300 effect in their decision-making neural responses in the gain context.

The behavioral results indicated that empathic concern can influence costly prosocial behaviors, and individuals give a higher rate of help to objects with high empathic concern. Empathy is a key prerequisite and motivation for prosocial, helpful, altruistic, and comforting behavior [41,45,46,47]. Moreover, increased individual empathic concern can lead to more prosocial behaviors [48]. The behavioral outcomes did not reveal an interaction between empathic concern and the situation. Researchers have found that Chinese participants exhibit strong in-group favoritism while pursuing fairness [49], indicating that even if they follow the principle of fairness, they will still exhibit stronger toward closer people. The manipulation of individuals’ empathy state in an experiment induced a greater overlap between others and themselves [50], making the individuals closer to others with high empathic concern. In this study, although individuals could also draw attention to fairness norms in the loss context, in-group favoritism caused by empathy led participants to make more prosocial decisions toward high-empathic-concern objects than low-empathic-concern objects.

ERP results indicate that high-empathic-concern objects induce larger P200 and P300 amplitudes than low-empathic-concern objects in the gain context, which is consistent with previous studies [41,51]. This finding indicates that empathy has a high level of motivational and emotional significance, resulting in a larger P200 and P300 amplitude. One study of generosity found that people tend to show stronger emotions toward individuals who induce empathy [52], further resulting in greater motivational and emotional significance and the allocation of more attentional resources. Empathy refers to experiencing other people’s psychological feelings through self-projection. When individuals treat other people’s emotional events as if they were their own emotional events, they produce a greater degree of emotional resonance response [47,53,54,55,56], thereby inducing a higher level of emotional and motivational significance. Self–other overlap refers to the incorporation of other people’s resources, perspectives, and traits into the self-concept [57]. Some studies have found that perspective-taking is an important way to improve self–other overlap, the mental process during which individuals imagine and speculate about the perspectives and attitudes of others, which is characteristic of empathy [58]. Many studies have also found that the higher the degree of overlap between self and target others, the easier it is for individuals to perform perspective-taking on others, and the more accurately they can understand the complex mental states of others, which produces higher emotional significance [59]. Neuroimaging studies have also shown that both empathic emotion induction and self-related stimulation can activate the ventral medial prefrontal cortex [60]. Therefore, in the gain context, high-empathic-concern conditions generate greater P200 and P300 than low-empathic-concern conditions.

Compared with the gain context, P200 and P300 did not show significant differences between the high- and low-empathic-concern conditions in the loss context. The inequality aversion model proposes that individuals pursue more fairness in the context of losses, which is a manifestation of strong reciprocity [18]. Implicit research also shows that individuals tend to associate loss with unfairness [16,17]. In the loss context, people are unwilling to cause losses to others and act on a fairness principle, implying that individuals with different levels of self-overlap have similar emotional and motivational significance when using P200 and P300 as indicators [23]. Under the implementation of fairness norms, individual preference for highly disadvantaged individuals disappears. Therefore, in the context of loss, the P200 and P300 wave amplitudes generated by the high- and low-empathic-concern conditions are similar. In addition, no effect of gender was found in any of the results, suggesting that gender did not play a significant role.

This study’s findings provide insights into how the brain processes interactions between empathy and loss-and-gain situations. As shown in the P200 and P300 results, contexts moderate the impact of empathic concern on prosocial decision-making. Empathic concern increases the motivation and emotional significance of prosocial decision-making in the gain context, but an individual’s execution of fairness norms in the loss context can explain the regulatory failure of empathic concern in prosocial decision-making.

## 5. Limitations

These results provide new directions for future research. First, this study only explores the impact of empathic concern on prosocial decision-making in the field of economic decision-making. In the future, we shall consider expanding the scope of prosocial behavior in a more realistic context. Second, the impact of different dimensions of empathy on prosocial behavior is inconsistent. Future research should explore the impact of empathy on prosocial behavior from multiple perspectives. Third, the relationship between empathy and prosocial behavior is related to many factors; in the future, other variables (such as moral beliefs) should be considered. Fourth, some studies suggest that prosocial decision-making in the context of loss is an intuitive response. Future research should consider incorporating time pressure and cognitive load to expand this study. Fifth, in this study, the influence of decisions on P300 cannot be considered due to insufficient trials under certain conditions. Future studies may consider the relationship between behavioral choice and P300. Lastly, Some studies have found that when the help object is a stranger, individuals have similar self–other overlap with different levels of empathy. This study used strangers to control participants’ self–other overlap, but it is possible that receivers with low-empathy conditions have more similar backgrounds to the subjects, leading to significance in self–other overlap. Future research should consider incorporating time pressure and cognitive load to expand on this study.

## 6. Conclusions

In summary, this study examines the impact of empathic concern on prosocial behavior in the gain and loss contexts and reveals the temporal dynamics of the underlying brain activity through ERP. The study’s results show that the effect of empathy on prosocial behavior is influenced by context. The P200 and P300 results indicate that empathic concern affects the cognitive evaluation of prosocial decision-making in the middle and later stages. In the gain context, prosocial decision-making is driven by the emotional motivation of the help object; however, the lack of P300 effects in the loss context is consistent with the inequality aversion model. This series of findings provides new insights into the prosocial decision-making behavior of individuals in specific contexts and helps us further understand how empathic concern modulates the process of prosocial decision-making.

## Data Availability

The data presented in this study are available on request from the corresponding author due to privacy.
